# Stress-Induced Functional Alterations in Amygdala: Implications for Neuropsychiatric Diseases

**DOI:** 10.3389/fnins.2018.00367

**Published:** 2018-05-29

**Authors:** Xin Zhang, Tong tong Ge, Guanghao Yin, Ranji Cui, Guoqing Zhao, Wei Yang

**Affiliations:** ^1^Jilin Provincial Key Laboratory on Molecular and Chemical Genetics, The Second Hospital of Jilin University, Changchun, China; ^2^Department of Anesthesiology, The Second Hospital of Jilin University, Changchun, China; ^3^Department of Anesthesiology, China-Japan Union Hospital of Jilin University, Changchun, China

**Keywords:** amygdala, stress, GABA, glutamate, neuropsychiatric diseases

## Abstract

The amygdala plays a major role in the processing of physiologic and behavioral responses to stress and is characterized by gamma-aminobutyric acid (GABA)-mediated high inhibitory tone under resting state. Human and animal studies showed that stress lead to a hyperactivity of amygdala, which was accompanied by the removal of inhibitory control. However, the contribution of hyperactivity of amygdala to stress-induced neuropsychiatric diseases, such as anxiety and mood disorders, is still dubious. In this review, we will summarize stress-induced various structural and functional alterations in amygdala, including the GABA receptors expression, GABAergic transmission and synaptic plasticity. It may provide new insight on the neuropathologic and neurophysiological mechanisms of neuropsychiatric diseases.

## Introduction

Stress is defined as a dynamic process to maintain organismal dynamic homeostasis, which is depicting the physiological and psychological responses of the organism to environmental stimulus. Various psychopathic diseases are generated while the organismal adaptive response is insufficient to sustain the basic homeostasis (Herbert, 1997; Bale, 2006). Thus, stress is an established and pivotal precipitating factor for several neuropsychiatric diseases, especially for anxiety, mood disorders and post-traumatic stress disorder (PTSD) (Koenigs and Grafman, 2009; Mahan and Ressler, 2012). Acute or chronic exposure to stress can lead to numerous long lasting adaptive changes in stress-susceptible brain regions, one of which exhibits entire disparate functional and structural alterations is the amygdala nuclei (Davidson et al., 2002; McEwen et al., 2016; Liu et al., 2017). Mounts of animal models indicate that the amygdala is hyperactivated or hyperreactive under stress state (Roozendaal et al., 1997; Pitman et al., 2012). Neuroimaging data also showed that patients with mood disorder always display an increased amygdalar activity (Bryant et al., 2008; Frodl et al., 2008).

Although the significant role of amygdalar activity in affecting stress-induced neuropsychiatric diseases is consistent, the specific molecular mechanisms and underlying neural circuitry are still obscure. Notably, GABA is one of the most important inhibitory neurotransmitters in the central nervous system (CNS) (Shiah and Yatham, 1998). Under resting state, the amygdala sustains a high GABAergic inhibitory control which contributes to the organism's resistance to various physiological and environmental stressors (Skórzewska et al., 2015). The hyperactivity and hyperresponsiveness of amygdala induced by stress is always accompanied by the removal of inhibitory control. Whether the impaired inhibitory control is the potential pathological cause of the generation of various neuropsychiatric diseases is still inconclusive. Therefore, in this article, we reviewed the current relatively studies and attempt to conclude the stress-induced neurotransmission alterations in amygdala and throw light on the potential therapeutic capacity in stress-related neuropsychiatric diseases.

## Stress-related neuropsychiatric diseases and amygdala

Neuropsychiatric illness is affected by several risk factors, including epigenetic factors, genetic factors, internal stimulus and environmental stimulus (Duman et al., 2016; Bartlett et al., 2017). Long-lasting stress condition induced by severe trauma, emotional arousal or stressful experiences appears to be one of the most prior inductive factors of several neuropsychiatric disorders, such as anxiety disorder, depression, schizophrenia, and PTSD (Yin et al., 2016). Common acute environmental stimuli for animal models comprise foot shock, restraint, social defeat and predator exposure (Fuchs, 2005; Hill et al., 2012). Acute exposure to these stimuli can lead to anxiety-like behavior (Sandi and Richter-Levin, 2009). Long lasting stress is a participating factor for anxiety and other mood disorders. Instead of physical stimuli mentioned above, fear conditioning models are selected to evaluate the persistence of traumatic memories and the extinction of fear (Mahan and Ressler, 2012). Arousal of traumatic memories is significantly associated with PTSD (Klein et al., 2003). Moreover, due to the hyperactivated hypothalamic pituitary adrenal (HPA) axis under stress situation, repeated management of glucocorticoid can be used as an experimental stress model (Angelier and Wingfield, 2013).

Clinical and animal studies showed that exposure to acute or chronic stress can induce morphological and functional changes in amygdala nuclei, which remarkably differ from that represented in the prefrontal cortex (PFC) and hippocampus. The predisposition of the amygdala to respond to emotional stimuli might influence the individual susceptibility to anxiety disorders. High anxious individuals are particularly more liable to process emotional information along with the hyperactivity of amygdala (Sandi and Richter-Levin, 2009).

## PFC-amygdala neuralcircuity in stress

Amounts of studies have confirmed the significant role of the PFC in integrating and processing sensory information (Zhuo, 2008; Miskovic and Keil, 2012; Harris and Mrsic-Flogel, 2013). The PFC can regulate the sensory or emotive stimuli-induced behavioral and physiological responses via complex connectional network with other brain structures (Negrón-Oyarzo et al., 2016). Human and animal studies have implicated the dysfunction of PFC-amygdala and PFC-hippocampus circuitry in the pathogenesis of stress-induced neuropsychiatric disorders (Kim et al., 2011; Duvarci and Pare, 2014). Earlier research suggests that stimulation of different subregions of the medial prefrontal cortex (mPFC) projections to the amygdala exerts respectively inhibitory or excitatory action on the central amygdala (CeA) neurons (Vidal-Gonzalez et al., 2006). Then, further research found that restraint stress was reported to induce an increment of 5-hydroxytryptamine (5-HT) output in the mPFC which subsequently promote GABA in the basolateral complex of amygdala (BLA) and selective 5-HT depletion in mPFC can decrease the stress-induced GABA release in BLA and attenuate the depression-like behavior (Andolina et al., 2013). These results indicate that the dysfunction of PFC-amygdala circuit might implicate in the stress-induced hyperreactivity of amygdala.

**Figure 1:**
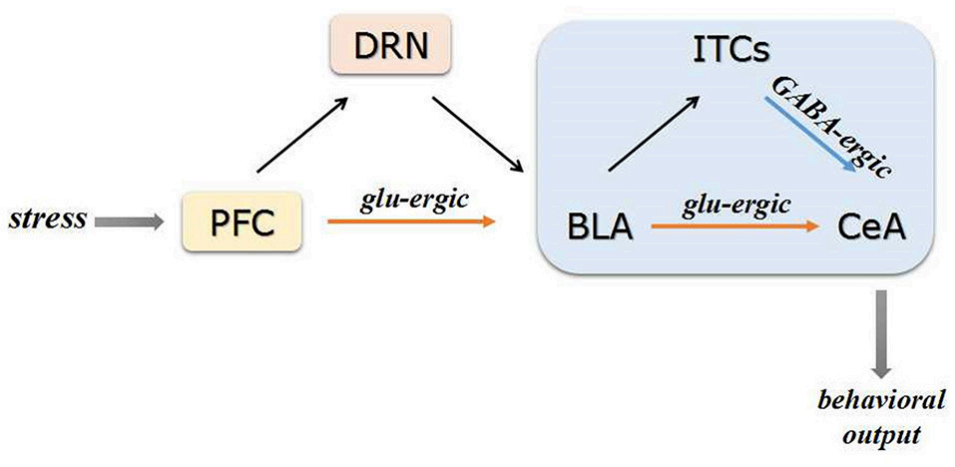
Schematic of PFC-BLA-CeA circuitry. The PFC transmits the sensory information to the BLA via amounts of glutamatergic projections. Subsequently, the glutamatergic BLA neurons directly project to the CeA or indirectly via GABAergic interneurons of ITCs. The PFC can affect BLA GABAergic system via regulating DRN 5-HT neurons under stress conditions. Projections from CeA mainly target to regions and brain circuits involved in physiological and behavioral responses to stress glu-ergic, glutamatergic.

The amygdala is a series of nuclei complex and usually divided into the BLA, the medial amygdala (MeA) and the CeA (Sah et al., 2003; Gilpin et al., 2015). The BLA can be further divided into lateral amygdala (LA) and basal amygdala (BA) (Pare and Duvarci, 2012). The existence of GABAergic interneurons and projections in amygdala complex is widely acknowledged and it's important to sustain the high inhibitory control over amygdala under resting state. As the major output nucleus of the amygdala, the CeA contains abundant local GABAergic interneurons and projections which can inhibit the other subordinated regions (Ehrlich et al., 2009). Morphological and physiological studies demonstrated that the intercalated cell clusters (ITCs) comprise a group of dense GABAergic neurons situated between the CeA and the BLA, which played an important role in the high inhibitory control over amygdala. The ITCs of amygdala can receive projections from BLA (de la Mora et al., 2010; Palomares-Castillo et al., 2012). As showed in Figure 1, the amygdala mainly receives sensory input from mPFC and subsequently integrates and transmits the signaling to other brain regions (McDonald, 1998). The ITCs can also directly receive projections from the mPFC (Duvarci and Pare, 2014). In addition, the dorsal raphe nucleus (DRN) 5-HT neurons were reported to participate in processing stress stimuli via regulating the neurotransmission in BLA (Christianson et al., 2010). A functional control of DRN 5-HT neurons by projection from mPFC was reported (Celada et al., 2002). Therefore, mPFC can also affect amygdala GABAergic system via regulating DRN 5-HT neurons.

Taken together, stress-activated mPFC neurons could directly project to BLA neurons and disrupt the GABAergic control in BLA, which contributes to the consequent inappropriate hyperactivation of downstream structures. Therefore, the mPFC might serve as a gate for sensory information transmission from BLA to CeA within amygdala microcircuitry. The CeA of amygdala could finally target to other regions involved in physiological and behavioral responses to stress (Akmaev et al., 2004; Pare and Duvarci, 2012).

## Stress, HPA axis activation and amygdala

The HPA axis is the central stress response system. Exposure to stress can significantly promote the activities of HPA axis and the synthesis of stress hormones (Menzaghi et al., 1993; Kalin et al., 1994; Heinrichs et al., 1995; Deppermann et al., 2014). Under long-lasting stress conditions, sustained hyperactivated HPA axis and the consequent high circulating glucocorticoids levels can lead to structural and functional disruption in amygdala via activating specific receptors (Gray and Bingaman, 1996). Reciprocally, the amygdala can strengthen the HPA activity via direct projections to the paraventricular nucleus of hypothalamus (PVN) (Davis and Shi, 1999).

Several animal studies have shown that administration of corticosterone into the CeA can potentiate anxiety-like behavior (Myers et al., 2005). Chronic administration of corticosterone can imitate the depressed effect of chronic stress on GABAergic tonic inhibition in LA. Such inhibition is mainly attributed to the activation of glucocorticoid receptor (GR) rather than mineralocorticoid receptor (MR) (Liu et al., 2014). Consistently, it was reported that chronic or acute administration of dexamethasone, a potent agonist of GR, can significantly cause a neurotransmission imbalance between glutamate and GABA via upregulation of GABAergic neurons and downregulation of glutamatergic neurons in amygdala (Wang et al., 2016). A substantial number of studies have confirmed the pivotal role of GR in the HPA axis regulation followed chronic stress (Furay et al., 2008). These findings suggest that stressors can lead to a hyperactivity of HPA axis and corticosterone release which both can despair the GABAergic control of the amygdala via activating GR, while the specific molecular mechanisms are stilled elusive.

Exposure to chronic stressors and stress-promoted corticosterone can upregulate the corticotropin releasing factor (CRF) expression in amygdala (Kalin et al., 1994; Shekhar et al., 2005). Pharmacological and genetic literatures have shown that CRF expression in amygdala can mediate the adequate behavioral responses to stress and increase the anxiety-like behaviors (Skórzewska et al., 2011; Regev et al., 2012; Callahan et al., 2013). Administration of a selective CRF1 receptor antagonist reversed the high corticosterone-induced anxiety-like behavior (Myers et al., 2005). Depression of CRF expression in CeA was found to mildly attenuate stress-induced behavioral abnormality (Regev et al., 2012; Callahan et al., 2013). On the one hand, GABA is the predominant co-transmitter in CRF neurons of amygdala (Gafford and Ressler, 2015). Several studies suggest that the anxiety-facilitated effects of CRF and CRF receptors partially depend on the interactions with GABA neurotransmission system in amygdala (Gray, 1993). It was found that administration of urocortin, a CRF1 and 2 receptors agonist, into the BLA promoted a long lasting anxiety-like behavior in rats, which showed a prominent reduction in both spontaneous and stimulation-evoked inhibitory postsynaptic currents (IPSCs) (Rainnie et al., 2004). Furthermore, it was found that application of exogenous CRF can significantly increase the mean frequency of GABAergic miniature inhibitory postsynaptic currents (mIPSC). The presynaptic GABA release might be promoted in the CeA of amygdala, which was associated with the kappa-opioid receptor (KOR) system in amygdala (Kang-Park et al., 2015). A recent research demonstrated that chronic, unpredictable stress increased the amplitude of evoked IPSCs and connectivity between CRF-expressing neurons in CeA (Partridge et al., 2016). These results suggest that stress-induced excessive CRF might induce a depression of local GABAergic inhibition and a resultant hyperexcitability of the amygdala. On the other hand, the CRF neurons in CeA of the amygdala can directly project to the PVN or via indirect GABAergic projections to the bed nucleus of the stria terminalis (BNST), which contribute to a further activation of HPA axis and CRF release (Davis and Shi, 1999). In addition, the MeA of amygdala can also lead to a disinhibition of PVN via GABAergic projection neurons (Jankord and Herman, 2008). Inversely, the basal HPA activity or the HPA axis responses to social interaction is not affected when the BLA is injured, indicating that the BLA and HPA axis might be weakly linked (Jankord and Herman, 2008).

## Amygdalar neurotransmission in regulation of stress

Other than the internal activation of HPA axis and excess secretion of stress hormones, short-term or long-term exposure to external stressors can induce hyperactivity of amygdala. Stress can induce various alterations of neurotransmission system in amygdala, mainly in GABA receptors adaption, the GABAergic inhibition and the synaptic neurotransmission. Lasting hyperactivity in amygdala might contribute to higher susceptibility to stress-related neuropsychiatric diseases. It was well known that the amygdala contains abundant local GABA interneurons and GABAergic projection neurons (Gilpin et al., 2015). Enduring high GABAergic inhibition in amygdala contributes to the resistance from various physiological and environmental stressors stimuli. Whether the impaired GABAergic control and hyperactivity in amygdala under stress exposure contribute to the pathogenesis of the neuropsychiatric diseases is worthy of investigation.

## Stress-induced phasic and tonic inhibition

Amygdala activation followed stress exposure is extensively reported in electrophysiology studies, while the majority of such studies focus on effect of single external stressor on the firing rate or action potentials in amygdala neurons. Few researches put emphasis on the long lasting stress animal models and their consequent anxiety- or depression-like behavior alterations.

Isoardi et al. demonstrated that acute restraint stress suppressed the IPSPs, which is generated by recurrent activation of GABAergic interneurons in BLA, contributing to a disinhibition of BLA pyramidal neurons (Isoardi et al., 2007). Consist with that, the BLA neurons of previously restrained animals preferred to be evoked and the evoked response can be normalized by a positive GABAergic modulator perfusion (Rodríguez Manzanares et al., 2005). These findings indicate that acute stress contributes to an impairment of evoked GABAergic inhibition in BLA.

A striking literature investigated the effects of both chronic immobilization and unpredictable stress on the GABAergic inhibitory modulation in amygdala. Instead of altering the GABAergic phasic inhibition, the most basic neurotransmission form via producing an inhibitory postsynaptic current in a “point-to-point” way (Mody, 2001), chronic stress was found to lead to a long-term loss of tonic GABAA receptor (GABAA-R) currents in the projection neurons in LA of amygdala (Liu et al., 2014). In contrast to the phasic inhibition, tonic inhibition is characterized by persistent inhibitory currents via continuously activation of extrasynaptic GABAA-Rs resulting from ambient GABA release (Carver and Reddy, 2013). GABAergic inhibitory control in BLA depends on both GABAergic phasic and tonic inhibition under resting state and the latter one contributes to sustaining a high threshold of activating the amygdala (Davern et al., 2014). A possible explanation for the unaffected phasic inhibition is that the impairment of phasic inhibition might occur instantly following exposure to stressor and recover in the stress-free phase. Furthermore, they also found that the long-lasting loss of tonic inhibition impaired the ability of GABA to suppress neuronal firing. Another interesting result was that long-term exposure (6–8 days) and daily 2 h exposure both reduced the tonic currents while no significance was detected in short-term exposure group and daily 15 min exposure group (Liu et al., 2014), indicating that the severity and duration of stressor exposure proportionally altered the tonic inhibition in LA. In addition, Aroniadou-Anderjaska1 et al. elucidated that activating the α_1A_ adrenoceptors located in GABAergic presynaptic terminals of BLA can facilitate GABA release, which contributes to the tonic inhibition of BLA pyramidal neurons under resting state (Aroniadou-Anderjaska et al., 2007). Notably, Rajbhandari et al. first reported that the noradrenergic (NE) α_1_ receptors and CRF1 receptors are colocalized on BLA glutamatergic projection neurons that innervate the nucleus accumbens (NAcc) (Rajbhandari et al., 2015). A long-lasting CRF1 receptors activation might induce a long lasting functional sensitization of the α_1_ receptors and subsequently have an effect on the GABAergic transmission of BLA (Rajbhandari et al., 2015). Taken together, acute or chronic exposure to external stressors can dampen the GABAergic inhibition of BLA in different manner through which both acute and chronic stress can overly activate the downstream brain regions and contribute to abnormal anxiogenic-like responses.

## Stress-induced GABA levels and GABA receptor alteration

A number of studies have reported that stress can induce GABA and GABA receptors alterations in amygdala (Sanders and Shekhar, 1995; Delaney and Sah, 1999). Accumulating evidence suggest that GABAA-R subunits in BLA of amygdala have been reported to mediate the anxiety in contextual fear conditioning (Jasnow and Huhman, 2001; Maren et al., 2001; Lin et al., 2009). Traumatic stress can also alter the expression of GABAA-R subunits (Ardi et al., 2016). An earlier research showed that juvenile stressor exposure increased the GABAA α2 and GABAA α3 subunits expression in amygdala (Jacobson-Pick and Richter-Levin, 2012). Whereas the opposite results exist, Tzanoulinou S et al. found that peripuberty stress induced a reduction of GABAA α3 expression in amygdala and peripubertally stressed mice displayed an increment of anxiety-like behaviors and impaired sociability at adulthood (Tzanoulinou et al., 2014). Another research showed that animals sustaining mild traumatic brain injury (mTBI) displayed anxiety-like behaviors, in the meanwhile the expression of α1, β2, and γ2 GABAA-Rs subunits decreased in BLA, which might relate to the acetylcholine receptor signaling pathway (Almeida-Suhett et al., 2014). These results suggest that stress-induced reduction of GABAA-Rs expression, indicating an impaired GABAergic transmission in amygdala, might affect the vulnerability to stress-induced behavioral abnormality. A research provides further support that high-anxiety rats sustaining repeated corticosterone treatment showed more anxiety-like behavior accompanying with a decrease in GABAA α2 subunit density in the CeA. Acute midazolam administration can significantly reverse the behavior impairment and increase GABAA α2 subunit density (Skórzewska et al., 2015). Different experimental conditions and procedure might explain the paradoxical results. Also, genetic factors, affecting the adaptive expression of GABAA-Rs followed stress, might result in the susceptivity to stress-induced neuropsychiatric diseases (Sarro et al., 2014). As we mentioned above, impaired extrasynaptic GABA receptors-mediated tonic inhibition contributes to the neuronal hyperactivity induced by chronic stress in BLA. The synaptic and extrasynaptic GABAA-Rs adapt to external and internal stressor stimuli in different manner (Jacob et al., 2008). The available studies mainly focus on the expression and sensitivity changes of GABAA-Rs followed stress and few of them discriminate these two types of receptors. Since the expression of GABAA-Rs displayed a tendency to be reduced followed stress exposure, this might explain the suppressed GABAergic inhibition in amygdala under stress conditions at molecular level.

Previous research has found that patients with mood disorder, particular depression and anxiety, had a lower GABA levels in plasma and cerebral spinal fluid than control (Petty and Sherman, 1984). Stress caused a reduction of glutamic acid decarboxylase (GAD) expression in amygdala, both in mRNA level and protein level in amygdala of stressed mice (Gilabert-Juan et al., 2011; Jacobson-Pick and Richter-Levin, 2012). However, opposite results showing that increased GABA levels in the venous blood of amygdala were also reported followed exposure to predator stress (Cook, 2004). Vivo microdialysis studies showed that acute restraint stress can increase GABA efflux in the BLA (Reznikov et al., 2009). Reagan et al. reported that acute restraint stress can increase the extracellular glutamate levels while have no effect on GABA levels in the BLA (Reagan et al., 2012). A recent research showed that restraint stress can induce an increment of 5-HT output in the mPFC and subsequently promote the GABA release in the BLA (Andolina et al., 2013). Taken together, due to the limitation of current experimental methods, the neurotransmitter level remains relatively low-sensitivity to be an indicator or biomarker to assess amygdala's response to different stressor stimuli. Contradictory results still exist and need to be further studied.

## Stress-induced synaptic plasticity changes

Lately, an emerging hypothesis revealed that the N-methyl-D-aspartate receptor (NMDAR)-dependent synaptic plasticity changes might implicate the pathogenesis of mood disorders (Gerhard et al., 2016). Thus it's appropriate to investigate the stress-induced synaptic plasticity alterations in amygdala and its interrelation with neuropsychiatric diseases. It was showed that the GluR1 expression and GluR1 phosphorylation of calcium-permeable α-amino-3-hydroxy-5-methyl-4-isoxazolepropionic acid receptors (CP-AMPARs) in BLA synaptosome were increased in a protein kinase A (PKA)-dependent manner after chronic stress exposure (Yi et al., 2017). Chronic stress-induced hypertrophy of BLA neuronal dendritic might contribute to an increment of AMPAR assemblies. It was well known that long-term AMPAR recruitment and removal are critical in the role of NMDAR-mediated long-term potentiation (LTP) (Cain, 1997; Paré, 2004). The LTP in GABA interneurons contributes to a feed-forward inhibition of principal neurons in BLA (Sharp, 2017). Although the behavioral evidence is still absent, the glutamate-mediated synaptic neurotransmission alterations appear to be involved in the potential mechanism of stress-induced hyperactivity of amygdala.

The endogenous cannabinoid (eCB) signaling can modulate both GABAergic and glutamatergic neurotransmission in BLA. Chronic restraint stress can reduce cannabinoid receptor type 1 (CB1)-mediated decrement of GABAergic synaptic transmission in BLA, in contrast with that, exposure to adverse acute stressors can cause a transient enhancement in short-term eCB signaling in the CeA (Ramikie and Patel, 2012). In our research, we demonstrated that exposure to single environmental stressor disrupted CB1-dependent long-term depression (LTD) within the BLA to CeA, while the NMDAR-dependent LTP and LTD of BLA to CeA synaptic transmission were not affected (Li et al., 2017). Pharmacology experiments showed that intra-CeA administration of CB1-selective antagonists prevented the acute stress-induced behavior impairment (Li et al., 2017). Thus, eCB-CB1 signaling-mediated synaptic alterations in BLA-CeA microcircuits of amygdala might play a critical role in the emotional processing following external stressors.

## Stress-induced amygdala morphological changes

The neuronal and synaptic alterations induced by stress appear to manifest as long-lasting morphological changes and structural adaption in amygdala, which might underlie the development of anxiety or other mood disorders (Kim et al., 2006; Roozendaal et al., 2009; Christoffel et al., 2011). It was found that after 21 days of stress-free recovery, chronic immobilization stress can even exhibit dendritic growth on spiny neurons of BLA, accompanying with enhanced long-lasting anxiety behaviors (Vyas et al., 2004). Vyas et al. had also reported the similar chronic immobilization stress-induced increment of dendritic arborization in pyramidal and stellate neurons of the BLA (Vyas et al., 2002). It was found that a single immobilization stress led to a gradual increment of spine density on principal neurons of the BLA accompanying with enhanced anxiety-like behavior. Such morphological and behavioral alterations showed up 10 days after the acute stress exposure (Mitra et al., 2005). Consist with that, it was found that exposure to 21 days restraint stress exhibited a reduced expression of neural cell adhesion molecule (PSA-NCAM) in CeA nuclei of rats (Cordero et al., 2005; Gilabert-Juan et al., 2011). Given that less dendritic arborization and spine density were detected in interneurons expressing PSA-NCAM than interneurons lacking PSA-NCAM, the chronic stress-triggered reduction of PSA-NCAM altered the functional connectivity of GABAergic interneurons and the dendritic reorganization of principal neurons in specific nucleus of amygdala (Gilabert-Juan et al., 2011). These results indicate that the generation of acute stress-induced synaptic alterations needs more time. However, chronic or repeated stress can instantly lead to a robust and persistent enhancement of spinogenesis and anxiety-like behaviors.

Stress causes reduction of brain derived neurotrophic factor (BDNF) expression and the mechanistic target of rapamycin (mTOR) in synapse, consequently contributing to the loss and atrophy of specific synapse in the brain regions implicated in depression, particularly the PFC and the hippocampus (Marsden, 2013; Abelaira et al., 2014; Vose and Stanton, 2017). Strikingly, the stress-induced BDNF expression in amygdala is inconsistent with that of PFC and hippocampus. Mounts of evidence have showed that the expression of BDNF in amygdala was increased after exposure to stress (Bennett and Lagopoulos, 2014). Opposite results exist that chronic unpredictable mild stress (CUMS)-treated rats showed a decrement of the expression of BDNF, postsynaptic density protein 95 (PSD-95) and synaptophysin in amygdala (Luo et al., 2013; Zhang et al., 2014). The PSD-95 and synaptophysin are well known as synapse-related proteins which can act as markers of synaptogenesis or synaptic potentiation. Furthermore, Yi et al. found that chronic restraint stress enhanced the expression of synaptophysin and PSD-95 at BLA synapses (Yi et al., 2017). Thus, the expression of BDNF in amygdala exhibited a tendency to increase followed restraint stress and this detected increment is in accordance with the promotion of spinogenesis in BLA induced by chronic stress

Together, chronic stress might induce synaptogenesis and synaptic incorporation in BLA synapses, while the precise relationship between the structural and functional synaptic changes of other nucleus of amygdala and the development of stress-related neuropsychiatric disease is still obscure. Thus, further evidence is needed to elucidate the deeper mechanisms.

## Discussion

Above all, three remarkable issues arise up and need further investigation. The first is the distinct regulatory role of different subregions within the amygdala. As mentioned above, the CeA and MeA nucleis both have GABAergic projections and can result in a disinhibition to PVN. While the link between BLA and HPA axis appears to be delicate, indicating that the BLA is insensitive to acute stress response. Furthermore, majority of electrophysiology studies illuminated that the neurons of BLA prefer to be evoked comparing with the neurons of CeA and other subregions of amygdala. The distinct role of these different subregions within the amygdala needs to be further investigated. The second issue is that whether the individual difference of GABAA-Rs adaption and morphological alterations induced by long lasting stress is affected by inherited genetic factors, which ultimately contribute to the individual susceptibility to the stress-induced neuropsychiatric diseases. The last one is the different pathological mechanisms of amygdala adapted to acute and chronic stress. Electrophysiology results indicated that acute stress could impair the phasic inhibition which occurred instantly following exposure to stressor and then recovered in the stress-free phase. In contrast to the acute stress, chronic stress was reported to impair the tonic inhibition in amygdala and consequently affect the ability of GABA to suppress neuronal firing. The morphological alterations of amygdala to acute stress can be accumulated. The effect of long lasting synaptic alterations induced by stress is manifested as synaptic morphological adaption. Significantly, the restructuring of dendrite and spines in amygdala is companied by stress-related behavior abnormalities. However, whether regulating the synaptic plasticity is involved in the underlying mechanisms of available antidepressant and anxiolytic therapy is still unclarified.

In conclusion, at the cellular and molecular level there are still inconsistent neurological alterations of the amygdala in various stress models. It's still argued that the hyperactivity and hyperresponsiveness of amygdala induced by stress is the primary predisposing factor of neuropsychiatric diseases. Although intra-amygdala administration of antidepressants or anxiolytic agents has been reported to inhibit the hyperactivity of amygdala and reverse the impairment of anxiety and depressive behaviors, the potential therapeutic property of amygdala needs to be deeply investigated. Future studies should take the severity and duration of stressor stimuli into consideration.

## Conclusions

Mounts of animal studies have shown that stress-related disorders, especially anxiety, depression and PTSD, are characterized by hyperactivity or hyperreactivity of the amygdala. It is well known that the amygdala plays a critical role in integrating sensory information. Amygdala can integrate the sensory information and subsequently transform them into behavioral output as a response to external stimuli through specific neural circuit. Besides, an important physiologic response to stress is the hyperactivity of the HPA axis, which is paralleled with the amygdala's response. The stress-induced HPA axis hyperactivity strengthens the amygdala through regulating the neuroendocrine system. In turn, the activated amygdala can reciprocally stimulate the neuronal projection to PVN.

The crucial alterations of stress-induced neurotransmission and synaptic plasticity in amygdala are intricate. The hyperactivity and hyperresponsiveness of amygdala followed stress attributes to the impaired GABAergic inhibition. Several critical neurotransmitters are involved, such as NE, cannabinoids and the CRF. The molecular mechanisms and potential role of stress-induced synaptic remodeling and increment of BDNF in specific amygdala nucleus are still obscure and need further investigations.

## Author contributions

XZ, TG, and GY wrote the manuscript. XZ, TG, GY, GZ, WY, and RC provided the critical revisions. All authors approved the final version of the manuscript for submission.

### Conflict of interest statement

The authors declare that the research was conducted in the absence of any commercial or financial relationships that could be construed as a potential conflict of interest.

## References

[B1] Abelaira H. M. Réus G. Z. Neotti M. V. Quevedo J. (2014). The role of mTOR in depression and antidepressant responses. Life Sci. 101, 10–14. 10.1016/j.lfs.2014.02.014 24582593

[B2] Akmaev I. G. Kalimullina L. B. Sharipova L. A. (2004). The central nucleus of the amygdaloid body of the brain: cytoarchitectonics, neuronal organization, connections. Neurosci. Behav. Physiol. 34, 603–610. 10.1023/B:NEAB.0000028292.14402.ad 15368908

[B3] Almeida-Suhett C. P. Prager E. M. Pidoplichko V. Figueiredo T. H. Marini A. M. Li Z.. (2014). Reduced GABAergic inhibition in the basolateral amygdala and the development of anxiety-like behaviors after mild traumatic brain injury. PLoS ONE 9:e102627. 10.1371/journal.pone.0102627 25047645 PMC4105413

[B4] Andolina D. Maran D. Valzania A. Conversi D. Puglisi-Allegra S. (2013). Prefrontal/amygdalar system determines stress coping behavior through 5-HT/GABA connection. Neuropsychopharmacology 38, 2057–2067. 10.1038/npp.2013.107 23636466 PMC3746690

[B5] Angelier F. Wingfield J. C. (2013). Importance of the glucocorticoid stress response in a changing world: theory, hypotheses and perspectives. Gen. Comp. Endocrinol. 190, 118–128. 10.1016/j.ygcen.2013.05.022 23770214

[B6] Ardi Z. Albrecht A. Richter-Levin A. Saha R. Richter-Levin G. (2016). Behavioral profiling as a translational approach in an animal model of posttraumatic stress disorder. Neurobiol. Dis. 88, 139–147. 10.1016/j.nbd.2016.01.012 26804028

[B7] Aroniadou-Anderjaska V. Qashu F. Braga M. F. (2007). Mechanisms regulating GABAergic inhibitory transmission in the basolateral amygdala: implications for epilepsy and anxiety disorders. Amino Acids 32, 305–315. 10.1007/s00726-006-0415-x 17048126

[B8] Bale T. L. (2006). Stress sensitivity and the development of affective disorders. Horm. Behav. 50, 529–533. 10.1016/j.yhbeh.2006.06.033 16901485

[B9] Bartlett A. A. Singh R. Hunter R. G. (2017). Anxiety and Epigenetics. Adv. Exp. Med. Biol. 978, 145–166. 10.1007/978-3-319-53889-1_8 28523545

[B10] Bennett M. R. Lagopoulos J. (2014). Stress and trauma: BDNF control of dendritic-spine formation and regression. Prog. Neurobiol. 112, 80–99. 10.1016/j.pneurobio.2013.10.005 24211850

[B11] Bryant R. A. Kemp A. H. Felmingham K. L. Liddell B. Olivieri G. Peduto A.. (2008). Enhanced amygdala and medial prefrontal activation during nonconscious processing of fear in posttraumatic stress disorder: an fMRI study. Hum. Brain Mapp. 29, 517–523. 10.1002/hbm.20415 17525984 PMC6870569

[B12] Cain D. P. (1997). LTP, NMDA, genes and learning. Curr. Opin. Neurobiol. 7, 235–242. 10.1016/S0959-4388(97)80012-8 9142751

[B13] Callahan L. B. Tschetter K. E. Ronan P. J. (2013). Inhibition of corticotropin releasing factor expression in the central nucleus of the amygdala attenuates stress-induced behavioral and endocrine responses. Front. Neurosci. 7:195. 10.3389/fnins.2013.00195 24194694 PMC3810776

[B14] Carver C. M. Reddy D. S. (2013). Neurosteroid interactions with synaptic and extrasynaptic GABA(A) receptors: regulation of subunit plasticity, phasic and tonic inhibition, and neuronal network excitability. Psychopharmacology 230, 151–188. 10.1007/s00213-013-3276-5 24071826 PMC3832254

[B15] Celada P. Puig M. V. Martín-Ruiz R. Casanovas J. M. Artigas F. (2002). Control of the serotonergic system by the medial prefrontal cortex: potential role in the etiology of PTSD and depressive disorders. Neurotox. Res. 4, 409–419. 10.1080/10298420290030550 12754155

[B16] Christianson J. P. Ragole T. Amat J. Greenwood B. N. Strong P. V. Paul E. D.. (2010). 5-hydroxytryptamine 2C receptors in the basolateral amygdala are involved in the expression of anxiety after uncontrollable traumatic stress. Biol. Psychiatry 67, 339–345. 10.1016/j.biopsych.2009.09.011 19914601 PMC3278236

[B17] Christoffel D. J. Golden S. A. Russo S. J. (2011). Structural and synaptic plasticity in stress-related disorders. Rev. Neurosci. 22, 535–549. 10.1515/RNS.2011.044 21967517 PMC3212803

[B18] Cook C. J. (2004). Stress induces CRF release in the paraventricular nucleus, and both CRF and GABA release in the amygdala. Physiol. Behav. 82, 751–762. 10.1016/j.physbeh.2004.06.013 15327926

[B19] Cordero M. I. Rodríguez J. J. Davies H. A. Peddie C. J. Sandi C. Stewart M. G. (2005). Chronic restraint stress down-regulates amygdaloid expression of polysialylated neural cell adhesion molecule. Neuroscience 133, 903–910. 10.1016/j.neuroscience.2005.03.046 15927407

[B20] Davern P. J. Chowdhury S. Jackson K. L. Nguyen-Huu T. P. Head G. A. (2014). GABAA receptor dysfunction contributes to high blood pressure and exaggerated response to stress in Schlager genetically hypertensive mice. J. Hypertens. 32, 352–362. 10.1097/HJH.0000000000000015 24270178

[B21] Davidson R. J. Pizzagalli D. Nitschke J. B. Putnam K. (2002). Depression: perspectives from affective neuroscience. Annu. Rev. Psychol. 53, 545–574. 10.1146/annurev.psych.53.100901.135148 11752496

[B22] Davis M. Shi C. (1999). The extended amygdala: are the central nucleus of the amygdala and the bed nucleus of the stria terminalis differentially involved in fear versus anxiety. Ann. N. Y. Acad. Sci. 877, 281–291. 10.1111/j.1749-6632.1999.tb09273.x 10415655

[B23] de la Mora M. P. Gallegos-Cari A. Arizmendi-García Y. Marcellino D. Fuxe K. (2010). Role of dopamine receptor mechanisms in the amygdaloid modulation of fear and anxiety: structural and functional analysis. Prog. Neurobiol. 90, 198–216. 10.1016/j.pneurobio.2009.10.010 19853006

[B24] Delaney A. J. Sah P. (1999). GABA receptors inhibited by benzodiazepines mediate fast inhibitory transmission in the central amygdala. J. Neurosci. 19, 9698–9704. 10.1523/JNEUROSCI.19-22-09698.1999 10559379 PMC6782952

[B25] Deppermann S. Storchak H. Fallgatter A. J. Ehlis A. C. (2014). Stress-induced neuroplasticity: (mal)adaptation to adverse life events in patients with PTSD–a critical overview. Neuroscience 283, 166–177. 10.1016/j.neuroscience.2014.08.037 25193848

[B26] Duman R. S. Aghajanian G. K. Sanacora G. Krystal J. H. (2016). Synaptic plasticity and depression: new insights from stress and rapid-acting antidepressants. Nat. Med. 22, 238–249. 10.1038/nm.4050 26937618 PMC5405628

[B27] Duvarci S. Pare D. (2014). Amygdala microcircuits controlling learned fear. Neuron 82, 966–980. 10.1016/j.neuron.2014.04.042 24908482 PMC4103014

[B28] Ehrlich I. Humeau Y. Grenier F. Ciocchi S. Herry C. Lüthi A. (2009). Amygdala inhibitory circuits and the control of fear memory. Neuron 62, 757–771. 10.1016/j.neuron.2009.05.026 19555645

[B29] Frodl T. Möller H. J. Meisenzahl E. (2008). Neuroimaging genetics: new perspectives in research on major depression. Acta Psychiatr. Scand. 118, 363–372. 10.1111/j.1600-0447.2008.01225.x 18644006

[B30] Fuchs E. (2005). Social stress in tree shrews as an animal model of depression: an example of a behavioral model of a CNS disorder. CNS Spectr. 10, 182–190. 10.1017/S1092852900010038 15744220

[B31] Furay A. R. Bruestle A. E. Herman J. P. (2008). The role of the forebrain glucocorticoid receptor in acute and chronic stress. Endocrinology 149, 5482–5490. 10.1210/en.2008-0642 18617609 PMC2584591

[B32] Gafford G. M. Ressler K. J. (2015). GABA and NMDA receptors in CRF neurons have opposing effects in fear acquisition and anxiety in central amygdala vs. bed nucleus of the stria terminalis. Horm Behav. 76, 136–142. 10.1016/j.yhbeh.2015.04.001 25888455 PMC4844457

[B33] Gerhard D. M. Wohleb E. S. Duman R. S. (2016). Emerging treatment mechanisms for depression: focus on glutamate and synaptic plasticity. Drug Discov. Today 21, 454–464. 10.1016/j.drudis.2016.01.016 26854424 PMC4803609

[B34] Gilabert-Juan J. Castillo-Gomez E. Pérez-Rando M. Moltó M. D. Nacher J. (2011). Chronic stress induces changes in the structure of interneurons and in the expression of molecules related to neuronal structural plasticity and inhibitory neurotransmission in the amygdala of adult mice. Exp. Neurol. 232, 33–40. 10.1016/j.expneurol.2011.07.009 21819983

[B35] Gilpin N. W. Herman M. A. Roberto M. (2015). The central amygdala as an integrative hub for anxiety and alcohol use disorders. Biol. Psychiatry 77, 859–869. 10.1016/j.biopsych.2014.09.008 25433901 PMC4398579

[B36] Gray T. S. (1993). Amygdaloid CRF pathways. Role in autonomic, neuroendocrine, and behavioral responses to stress. Ann. N. Y. Acad. Sci. 697, 53–60. 10.1111/j.1749-6632.1993.tb49922.x 8257022

[B37] Gray T. S. Bingaman E. W. (1996). The amygdala: corticotropin-releasing factor, steroids, and stress. Crit. Rev. Neurobiol. 10, 155–168. 10.1615/CritRevNeurobiol.v10.i2.10 8971127

[B38] Harris K. D. Mrsic-Flogel T. D. (2013). Cortical connectivity and sensory coding. Nature. 503, 51–58. 10.1038/nature12654 24201278

[B39] Heinrichs S. C. Menzaghi F. Merlo Pich E. Britton K. T. Koob G. F. (1995). The role of CRF in behavioral aspects of stress. Ann. N. Y. Acad. Sci. 771, 92–104. 10.1111/j.1749-6632.1995.tb44673.x 8597448

[B40] Herbert J. (1997). Fortnighly review. Stress, the brain, and mental illness. BMJ 315, 530–535. 10.1136/bmj.315.7107.530 9329310 PMC2127355

[B41] Hill M. N. Hellemans K. G. Verma P. Gorzalka B. B. Weinberg J. (2012). Neurobiology of chronic mild stress: parallels to major depression. Neurosci. Biobehav. Rev. 36, 2085–2117. 10.1016/j.neubiorev.2012.07.001 22776763 PMC4821201

[B42] Isoardi N. A. Bertotto M. E. Martijena I. D. Molina V. A. Carrer H. F. (2007). Lack of feedback inhibition on rat basolateral amygdala following stress or withdrawal from sedative-hypnotic drugs. Eur. J. Neurosci. 26, 1036–1044. 10.1111/j.1460-9568.2007.05714.x 17666080

[B43] Jacob T. C. Moss S. J. Jurd R. (2008). GABA(A) receptor trafficking and its role in the dynamic modulation of neuronal inhibition. Nat. Rev. Neurosci. 9, 331–343. 10.1038/nrn2370 18382465 PMC2709246

[B44] Jacobson-Pick S. Richter-Levin G. (2012). Short- and long-term effects of juvenile stressor exposure on the expression of GABAA receptor subunits in rats. Stress 15, 416–424. 10.3109/10253890.2011.634036 22044189

[B45] Jankord R. Herman J. P. (2008). Limbic regulation of hypothalamo-pituitary-adrenocortical function during acute and chronic stress. Ann. N. Y. Acad. Sci. 1148, 64–73. 10.1196/annals.1410.012 19120092 PMC2637449

[B46] Jasnow A. M. Huhman K. L. (2001). Activation of GABA(A) receptors in the amygdala blocks the acquisition and expression of conditioned defeat in Syrian hamsters. Brain Res. 920, 142–150. 10.1016/S0006-8993(01)03054-2 11716820

[B47] Kalin N. H. Takahashi L. K. Chen F. L. (1994). Restraint stress increases corticotropin-releasing hormone mRNA content in the amygdala and paraventricular nucleus. Brain Res. 656, 182–186. 10.1016/0006-8993(94)91382-X 7804835

[B48] Kang-Park M. Kieffer B. L. Roberts A. J. Siggins G. R. Moore S. D. (2015). Interaction of CRF and kappa opioid systems on GABAergic neurotransmission in the mouse central amygdala. J. Pharmacol. Exp. Ther. 355, 206–211. 10.1124/jpet.115.225870 26350161 PMC4613963

[B49] Kim J. J. Song E. Y. Kosten T. A. (2006). Stress effects in the hippocampus: synaptic plasticity and memory. Stress 9, 1–11. 10.1080/10253890600678004 16753928

[B50] Kim M. J. Loucks R. A. Palmer A. L.. (2011). The structural and functional connectivity of the amygdala: from normal emotion to pathological anxiety. Behav. Brain Res. 223, 403–410. 10.1016/j.bbr.2011.04.025 21536077 PMC3119771

[B51] Klein E. Caspi Y. Gil S. (2003). The relation between memory of the traumatic event and PTSD: evidence from studies of traumatic brain injury. Can. J. Psychiatry 48, 28–33. 10.1177/070674370304800106 12635561

[B52] Koenigs M. Grafman J. (2009). Posttraumatic stress disorder: the role of medial prefrontal cortex and amygdala. Neuroscientist 15, 540–548. 10.1177/1073858409333072 19359671 PMC2771687

[B53] Li B. Ge T. Cui R. (2017). Long-term plasticity in amygdala circuits: implication of CB1-dependent LTD in stress. Mol Neurobiol. 55, 4107–4114. 10.1007/s12035-017-0643-y 28593436

[B54] Lin H. C. Mao S. C. Gean P. W. (2009). Block of gamma-aminobutyric acid-A receptor insertion in the amygdala impairs extinction of conditioned fear. Biol. Psychiatry 66, 665–673. 10.1016/j.biopsych.2009.04.003 19482263

[B55] Liu W. Ge T. Leng Y. Pan Z. Fan J. Yang W.. (2017). The role of neural plasticity in depression: from hippocampus to prefrontal cortex. Neural Plast. 2017:6871089. 10.1155/2017/6871089 28246558 PMC5299163

[B56] Liu Z. P. Song C. Wang M. He Y. Xu X. B. Pan H. Q.. (2014). Chronic stress impairs GABAergic control of amygdala through suppressing the tonic GABAA receptor currents. Mol. Brain 7:32. 10.1186/1756-6606-7-32 24758222 PMC4012764

[B57] Luo J. Zhang L. Ning N. Jiang H. Yu S. Y. (2013). Neotrofin reverses the effects of chronic unpredictable mild stress on behavior via regulating BDNF, PSD-95 and synaptophysin expression in rat. Behav. Brain Res. 253, 48–53. 10.1016/j.bbr.2013.07.014 23850356

[B58] Mahan A. L. Ressler K. J. (2012). Fear conditioning, synaptic plasticity and the amygdala: implications for posttraumatic stress disorder. Trends Neurosci. 35, 24–35. 10.1016/j.tins.2011.06.007 21798604 PMC3206195

[B59] Maren S. Yap S. A. Goosens K. A. (2001). The amygdala is essential for the development of neuronal plasticity in the medial geniculate nucleus during auditory fear conditioning in rats. J. Neurosci. 21:RC135. 10.1523/JNEUROSCI.21-06-j0001.2001 11245704 PMC6762621

[B60] Marsden W. N. (2013). Synaptic plasticity in depression: molecular, cellular and functional correlates. Prog. Neuropsychopharmacol. Biol. Psychiatry 43, 168–184. 10.1016/j.pnpbp.2012.12.012 23268191

[B61] McDonald A. J. (1998). Cortical pathways to the mammalian amygdala. Prog. Neurobiol. 55, 257–332. 10.1016/S0301-0082(98)00003-3 9643556

[B62] McEwen B. S. Nasca C. Gray J. D. (2016). Stress effects on neuronal structure: hippocampus, amygdala, and prefrontal cortex. Neuropsychopharmacology 41, 3–23. 10.1038/npp.2015.171 26076834 PMC4677120

[B63] Menzaghi F. Heinrichs S. C. Pich E. M. Weiss F. Koob G. F. (1993). The role of limbic and hypothalamic corticotropin-releasing factor in behavioral responses to stress. Ann. N. Y. Acad. Sci. 697, 142–154. 10.1111/j.1749-6632.1993.tb49929.x 8257007

[B64] Miskovic V. Keil A. (2012). Acquired fears reflected in cortical sensory processing: a review of electrophysiological studies of human classical conditioning. Psychophysiology 49, 1230–1241. 10.1111/j.1469-8986.2012.01398.x 22891639 PMC3422776

[B65] Mitra R. Jadhav S. McEwen B. S. Vyas A. Chattarji S. (2005). Stress duration modulates the spatiotemporal patterns of spine formation in the basolateral amygdala. Proc. Natl. Acad. Sci. U.S.A. 102, 9371–9376. 10.1073/pnas.0504011102 15967994 PMC1166638

[B66] Mody I. (2001). Distinguishing between GABA(A) receptors responsible for tonic and phasic conductances. Neurochem. Res. 26, 907–913. 10.1023/A:1012376215967 11699942

[B67] Myers D. A. Gibson M. Schulkin J. Greenwood Van-Meerveld B. (2005). Corticosterone implants to the amygdala and type 1 CRH receptor regulation: effects on behavior and colonic sensitivity. Behav. Brain Res. 161, 39–44. 10.1016/j.bbr.2005.03.001 15904708

[B68] Negrón-Oyarzo I. Aboitiz F. Fuentealba P. (2016). Impaired functional connectivity in the prefrontal cortex: a mechanism for chronic stress-induced neuropsychiatric disorders. Neural Plast. 2016:7539065. 10.1155/2016/7539065 26904302 PMC4745936

[B69] Palomares-Castillo E. Hernández-Pérez O. R. Pérez-Carrera D. Crespo-Ramírez M. Fuxe K. de la Mora M. P. (2012). The intercalated paracapsular islands as a module for integration of signals regulating anxiety in the amygdala. Brain Res. 1476, 211–234. 10.1016/j.brainres.2012.03.047 22516107

[B70] Paré D. (2004). Presynaptic induction and expression of NMDA-dependent LTP. Trends Neurosci. 27, 440–441. 10.1016/j.tins.2004.05.004 15271488

[B71] Pare D. Duvarci S. (2012). Amygdala microcircuits mediating fear expression and extinction. Curr. Opin. Neurobiol. 22, 717–723. 10.1016/j.conb.2012.02.014 22424846 PMC3380167

[B72] Partridge J. G. Forcelli P. A. Luo R.. (2016). Stress increases GABAergic neurotransmission in CRF neurons of the central amygdala and bed nucleus stria terminalis. Neuropharmacology 107, 239–250. 10.1016/j.neuropharm.2016.03.029 27016019 PMC7025394

[B73] Petty F. Sherman A. D. (1984). Plasma GABA levels in psychiatric illness. J. Affect. Disord. 6, 131–138. 10.1016/0165-0327(84)90018-1 6233345

[B74] Pitman R. K. Rasmusson A. M. Koenen K. C.. (2012). Biological studies of post-traumatic stress disorder. Nat. Rev. Neurosci. 13, 769–787. 10.1038/nrn3339 23047775 PMC4951157

[B75] Rainnie D. G. Bergeron R. Sajdyk T. J. Patil M. Gehlert D. R. Shekhar A. (2004). Corticotrophin releasing factor-induced synaptic plasticity in the amygdala translates stress into emotional disorders. J. Neurosci. 24, 3471–3479. 10.1523/JNEUROSCI.5740-03.2004 15071094 PMC6729749

[B76] Rajbhandari A. K. Baldo B. A. Bakshi V. P. (2015). Predator stress-induced CRF release causes enduring sensitization of basolateral amygdala norepinephrine systems that promote PTSD-like startle abnormalities. J. Neurosci. 35, 14270–14285. 10.1523/JNEUROSCI.5080-14.2015 26490866 PMC4683687

[B77] Ramikie T. S. Patel S. (2012). Endocannabinoid signaling in the amygdala: anatomy, synaptic signaling, behavior, and adaptations to stress. Neuroscience 204, 38–52. 10.1016/j.neuroscience.2011.08.037 21884761 PMC3236282

[B78] Reagan L. P. Reznikov L. R. Evans A. N. Gabriel C. Mocaër E. Fadel J. R. (2012). The antidepressant agomelatine inhibits stress-mediated changes in amino acid efflux in the rat hippocampus and amygdala. Brain Res. 1466, 91–98. 10.1016/j.brainres.2012.05.039 22647752

[B79] Regev L. Tsoory M. Gil S. Chen A. (2012). Site-specific genetic manipulation of amygdala corticotropin-releasing factor reveals its imperative role in mediating behavioral response to challenge. Biol. Psychiatry 71, 317–326. 10.1016/j.biopsych.2011.05.036 21783178

[B80] Reznikov L. R. Reagan L. P. Fadel J. R. (2009). Effects of acute and repeated restraint stress on GABA efflux in the rat basolateral and central amygdala. Brain Res. 1256, 61–68. 10.1016/j.brainres.2008.12.022 19124010

[B81] Rodríguez Manzanares P. A. Isoardi N. A. Carrer H. F. Molina V. A. (2005). Previous stress facilitates fear memory, attenuates GABAergic inhibition, and increases synaptic plasticity in the rat basolateral amygdala. J. Neurosci. 25, 8725–8734. 10.1523/JNEUROSCI.2260-05.2005 16177042 PMC6725501

[B82] Roozendaal B. Koolhaas J. M. Bohus B. (1997). The role of the central amygdala in stress and adaption. Acta Physiol. Scand. Suppl. 640, 51–54. 9401606

[B83] Roozendaal B. McEwen B. S. Chattarji S. (2009). Stress, memory and the amygdala. Nat. Rev. Neurosci. 10, 423–433. 10.1038/nrn2651 19469026

[B84] Sah P. Faber E. S. Lopez De Armentia M. Power J. (2003). The amygdaloid complex: anatomy and physiology. Physiol. Rev. 83, 803–834. 10.1152/physrev.00002.2003 12843409

[B85] Sanders S. K. Shekhar A. (1995). Regulation of anxiety by GABAA receptors in the rat amygdala. Pharmacol. Biochem. Behav. 52, 701–706. 10.1016/0091-3057(95)00153-N 8587908

[B86] Sandi C. Richter-Levin G. (2009). From high anxiety trait to depression: a neurocognitive hypothesis. Trends Neurosci. 32, 312–320. 10.1016/j.tins.2009.02.004 19409624

[B87] Sarro E. C. Sullivan R. M. Barr G. (2014). Unpredictable neonatal stress enhances adult anxiety and alters amygdala gene expression related to serotonin and GABA. Neuroscience 258, 147–161. 10.1016/j.neuroscience.2013.10.064 24240029 PMC4050971

[B88] Sharp B. M. (2017). Basolateral amygdala and stress-induced hyperexcitability affect motivated behaviors and addiction. Transl. Psychiatry 7:e1194. 10.1038/tp.2017.161 28786979 PMC5611728

[B89] Shekhar A. Truitt W. Rainnie D. Sajdyk T. (2005). Role of stress, corticotrophin releasing factor (CRF) and amygdala plasticity in chronic anxiety. Stress 8, 209–219. 10.1080/10253890500504557 16423710

[B90] Shiah I. S. Yatham L. N. (1998). GABA function in mood disorders: an update and critical review. Life Sci. 63, 1289–1303. 10.1016/S0024-3205(98)00241-0 9768867

[B91] Skórzewska A. Bidzinski A. Lehner M. Turzynska D. Sobolewska A. Wisłowska-Stanek A.. (2011). The localization of brain sites of anxiogenic-like effects of urocortin-2. Neuropeptides 45, 83–92. 10.1016/j.npep.2010.11.003 21168912

[B92] Skórzewska A. Lehner M. Wisłowska-Stanek A.. (2015). GABAergic control of the activity of the central nucleus of the amygdala in low- and high-anxiety rats. Neuropharmacology 99, 566–576. 10.1016/j.neuropharm.2015.08.039 26318100

[B93] Tzanoulinou S. García-Mompó C. Castillo-Gómez E. Veenit V. Nacher J. Sandi C. (2014). Long-term behavioral programming induced by peripuberty stress in rats is accompanied by GABAergic-related alterations in the Amygdala. PLoS ONE 9:e94666. 10.1371/journal.pone.0094666 24736324 PMC3988094

[B94] Vidal-Gonzalez I. Vidal-Gonzalez B. Rauch S. L. Quirk G. J. (2006). Microstimulation reveals opposing influences of prelimbic and infralimbic cortex on the expression of conditioned fear. Learn. Mem. 13, 728–733. 10.1101/lm.306106 17142302 PMC1783626

[B95] Vose L. R. Stanton P. K. (2017). Synaptic plasticity, metaplasticity and depression. Curr. Neuropharmacol. 15, 71–86. 10.2174/1570159X14666160202121111 26830964 PMC5327460

[B96] Vyas A. Mitra R. Shankaranarayana Rao B. S. Chattarji S. (2002). Chronic stress induces contrasting patterns of dendritic remodeling in hippocampal and amygdaloid neurons. J. Neurosci. 22, 6810–6818. 10.1523/JNEUROSCI.22-15-06810.2002 12151561 PMC6758130

[B97] Vyas A. Pillai A. G. Chattarji S. (2004). Recovery after chronic stress fails to reverse amygdaloid neuronal hypertrophy and enhanced anxiety-like behavior. Neuroscience 128, 667–673. 10.1016/j.neuroscience.2004.07.013 15464275

[B98] Wang G. Y. Zhu Z. M. Cui S. Wang J. H. (2016). Glucocorticoid induces incoordination between Glutamatergic and GABAergic neurons in the amygdala. PLoS ONE 11:e0166535. 10.1371/journal.pone.0166535 27861545 PMC5115758

[B99] Yi E. S. Oh S. Lee J. K. Leem Y. H. (2017). Chronic stress-induced dendritic reorganization and abundance of synaptosomal PKA-dependent CP-AMPA receptor in the basolateral amygdala in a mouse model of depression. Biochem. Biophys. Res. Commun. 486, 671–678. 10.1016/j.bbrc.2017.03.093 28336441

[B100] Yin X. Guven N. Dietis N. (2016). Stress-based animal models of depression: Do we actually know what we are doing. Brain Res. 1652, 30–42. 10.1016/j.brainres.2016.09.027 27663969

[B101] Zhang L. Luo J. Zhang M. Yao W. Ma X. Yu S. Y (2014). Effects of curcumin on chronic, unpredictable, mild, stress-induced depressive-like behaviour and structural plasticity in the lateral amygdala of rats. Int. J. Neuropsychopharmacol. 17, 793–806. 10.1017/S1461145713001661 24405689

[B102] Zhuo M. (2008). Cortical excitation and chronic pain. Trends Neurosci. 31, 199–207. 10.1016/j.tins.2008.01.003 18329111

